# A universal growth limit for circular lichens

**DOI:** 10.1098/rsif.2018.0063

**Published:** 2018-06-06

**Authors:** Agnese Seminara, Joerg Fritz, Michael P. Brenner, Anne Pringle

**Affiliations:** 1 CNRS, Université Côte d'Azur, Institut de Physique de Nice, UMR7010, Parc Valrose 06108, Nice, France; 2 School of Engineering and Applied Sciences, Harvard University, Cambridge, MA, USA; 3 Department of Botany, University of Wisconsin-Madison, Madison, WI, USA; 4 Department of Bacteriology, University of Wisconsin-Madison, Madison, WI, USA

**Keywords:** coffee drop effect, fungi, growth rate, lichenometry, microbiology, population biology

## Abstract

Lichens fix carbon dioxide from the air to build biomass. Crustose and foliose lichens grow as nearly flat, circular disks. Smaller individuals grow slowly, but with small, steady increases in radial growth rate over time. Larger individuals grow more quickly and with a roughly constant radial velocity maintained over the lifetime of the lichen. We translate the coffee drop effect to model lichen growth and demonstrate that growth patterns follow directly from the diffusion of carbon dioxide in the air around a lichen. When a lichen is small, carbon dioxide is fixed across its surface, and the entire thallus contributes to radial growth, but when a lichen is larger carbon dioxide is disproportionately fixed at the edges of an individual, which are the primary drivers of growth. Tests of the model against data suggest it provides an accurate, robust, and universal framework for understanding the growth dynamics of both large and small lichens in nature.

## 1. Introduction

Lichens are symbioses of fungi and photosynthetic algae or bacteria, and are ubiquitous on our planet [1,2], found at the poles, in boreal, temperate and tropical forests, and in deserts and other biomes. They grow on rocks, bark or leaves, soil, and other substrates, weathering the rock, stabilizing soil and providing animals with food, shelter and camouflage. In the environment, lichens are important carbon and nitrogen sinks [3,4], dominating approximately 8% of Earth's terrestrial ecosystems [1].

Lichens grow slowly, and may become very old. Experimental data on the growth rates of crustose and foliose lichens, which are close to circular disks when mature [5], suggest many species grow with similar dynamics. Small lichens grow slowly, but at a steadily increasing rate; growth rates level to a constant as individuals reach a larger size. Despite the variability inherent in ecological data taken from nature [6], this pattern is generally observed and is supported by the field of lichenometry, which uses lichens to date geological events [7], for example rockfalls [8], by using thallus size and measured or extrapolated growth rates to calculate an age of the substrate. Although lichenometry is a popular technique [9], the forces shaping the growth rates of lichens remain unclear [10,11].

Various models have been developed to describe lichen growth, with the most successful emphasizing the fixation and movement of carbon within a thallus [12–14]. The models assume atmospheric carbon is fixed uniformly over the surface of a thallus, and that *internal transport* of carbon to the edge causes radial expansion. Qualitatively, these assumptions reproduce observed patterns of growth: when the lichen is small the entire structure contributes to carbon flux towards the edge, hence growth rates initially increase with the area of the lichen. But above a critical size, internal transport within the lichen cannot keep up with growth; expansion reaches a steady state where only a fixed band close to the outer edge contributes to carbon flux and biomass growth.

Tests of the models' assumptions are difficult and depend on detailed knowledge of carbon fixation, respiration and carbon flux within a thallus. A thorough discussion of what is known and not known about the carbon economy of lichens is provided by [15]. To date, models have estimated the different parameters by fitting predictions to available growth rate data [13,14].

**Figure 1.**
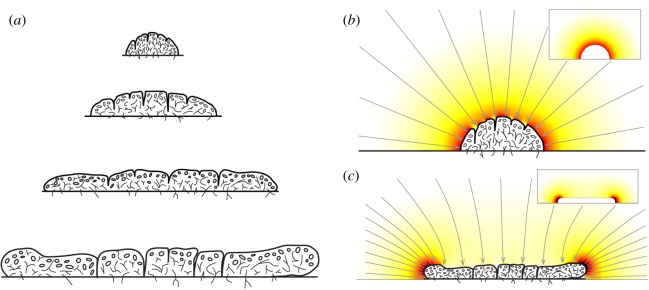
Lichen growth and associated flow patterns for different thallus sizes. (*a*) Cross section through a typical, growing lichen at different time points, adapted with permission from ref. [17], page 9. Radial growth continues even after change in H slows, and at maturity the morphology of the thallus changes from a more rounded to a disk-like shape. (*b*) The diffusion pattern around a small lichen creates a uniform flux over the entire surface area. Colour represents magnitude of flux *q* from 0 (white) to maximum on the surface of the lichen (red). Grey arrows represent streamlines along which the carbon is transported towards the lichen. Their density is proportional to flux *q*. (Inset) Solution of equation (2.1) towards a perfectly smooth hemispherical lichen. (*c*) The diffusion pattern around a larger lichen is distinctly different, with most of the carbon flux concentrated at the edges of a thallus. Colour represents flux *q* and grey arrows streamlines, just as in panel (*b*). (Inset) Solution of equation (2.1) towards the perfectly smooth version of the corrugated lichen in the main panel. The solutions in the main panels (*b*,*c*) agree very well with those shown in the insets, as the details of lichen surface are smoothed out by the Laplace equation. Diffusive fluxes in (*b*) and (*c*) are computed through finite element simulations of equation (2.1) in the three-dimensional space above the lichen. The volume of integration is a large (effectively infinite) cylinder; the three-dimensional shapes of the lichen are obtained by rotation of the cross sections (top and bottom of panel (*a*) for simulations in (*b*) and (*c*) respectively; their perfectly smooth replicas in the insets). We used the Laplace equation module of COMSOL Multiphysics on a physics-controlled triangular mesh. Boundary conditions are: *c* = *c*_∞_ at the top and lateral boundaries of the integration volume; no flux at the lower boundary and *c* = 0 at lichen surface. (Online version in colour.)

We propose a simpler model of lichen growth, based on a previously overlooked, fundamental fluid mechanical constraint on carbon flux. The model predicts a universal limit to lichen growth as a direct consequence of the diffusion of carbon dioxide in air, with no assumptions about the specific nature of metabolic rates or carbon movement inside a lichen. The central idea is adapted from a widely known study of stains left by evaporating coffee drops [16]. Stains take a specific pattern because of the evaporation of water from the coffee drop, and in our model, absorption of carbon dioxide by the lichen takes the same role in lichen growth as water evaporation does in the coffee drop problem. As carbon is fixed by a thallus, the carbon dioxide in the air close to the lichen surface is depleted. Depletion causes an uneven diffusive flux of carbon dioxide towards the lichen, stronger near the edges versus at the centre of a lichen (figure 1). This mechanism, considered by itself, reproduces the saturation of the growth rate, even if the internal transport of carbon within a lichen is entirely neglected. The model leads to a quantitative prediction for the expansion rate of large lichens, that depends only on the diffusion constant *D* of CO_2_ in air; the density of carbon as CO_2_ in air *ρ*_air_, the density of carbon in the biomass *ρ*_lichen_, the height *H* of the lichen, and the fraction of time the lichen is photosynthetically active, *e*.

We present data collected for a population of *Xanthoparmelia* lichens growing in Petersham, MA, USA, and revisit published data of lichens in other genera. Data provide empirical support for our model, moreover, the data of all species collapse onto a single growth curve, suggesting the growth dynamics of small lichens are also governed by universal principles. We extend our model assuming that, even for small lichens, carbon dioxide flux is the limiting growth factor. Height, a rarely studied feature of the growth dynamics of circular lichens, emerges as the parameter critical to reproducing experimental data. Moreover, the model implies newly established lichens will grow as spherical balls, a result consistent with our observations. The model suggests new experiments targeting both the shape and ultimate height of young lichens: if our hypothesis is correct, the height and shape of a developing lichen must follow the specific pattern predicted by our model. Although height and shape are measurable parameters, and appear critical to the growth dynamics of lichens, they have been previously overlooked in the study of lichen growth.

## 2. Diffusion of CO_2_ above the lichen enforces an upper limit for growth speed

Because the air is nearly still within the boundary layer close to the lichen, advection can be neglected and at steady state, the concentration of carbon dioxide, *c*, obeys the Laplace equation2.1
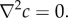
The density of photobionts is greatest close to the surface of most lichens, and at a steady state CO_2_ will be completely depleted very near the surface of a thallus. We thus impose absorption boundary conditions, i.e. that *c*_lichen_ = 0 at the surface of the lichen. Far away from the lichen, at the edge of the boundary layer, *c*(∞) = *c*_CO_2__, where *c*_CO_2__ is the average concentration of CO_2_ in free stream air. The condition of a boundary layer of nearly still air above a lichen, with a thickness larger than the other length scales in the problem, is satisfied in most environments. Although lichens may live outside of canopies where wind may be strong, natural terrain is never perfectly flat. Typical values of roughness are larger than the height of a lichen even for level terrain with no vegetation [18], thus the mean wind close to a lichen is likely negligible in most cases. Fluctuations of wind velocity would affect carbon dioxide transport by modifying the diffusion constant.

The mass flux *q* of carbon per unit area into the lichen is *q* = − *M*_c_*D*∇*c*, where *M*_c_ is the molar mass of carbon. We assume that the flux into the substrate the lichen is growing on, for example a rock, is zero. The Laplace equation with this type of boundary condition is a classic problem in physics, and its solutions are well understood. Originally encountered in electrodynamics, where *c* would be an electric potential and *q* the field strength on the surface of an ideal conductor, the problem is also encountered in fluid dynamics, for example in the evaporation of droplets, a problem nearly identical to the present problem except that the direction of the flux is reversed [16,19]. The pattern of diffusion over an absorber depends on its shape: in the context of lichens, we are interested in two asymptotic limits with respect to the two length scales of the thallus, height *H* and circular radius *R*. A schematic cross section of a typical, growing lichen, taken from an authoritative source [17], illustrates the two limits (figure 1). Large lichens (*R* > *H*) resemble circular disks while small lichens (*R* < *H*) are more rounded. Numerical solutions of equation (2.1) obtained with finite element simulations using COMSOL Multiphysics (see figure 1 caption) demonstrate the two distinctly different patterns of carbon dioxide flux toward a small versus a large lichen (figure 1*b*,*c*).

## 3. The growth rate for large lichens

When the lichen is large, its morphology resembles a flat, circular disk (figure 1). For a disk the flux is non-uniform in the radial direction *r* and very high at the edge3.1
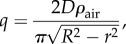
where *ρ*_air_ = *M*_c_*c*_CO_2__. Equation (3.1) corresponds to the electric field close to a circular conducting disc, see [20] §3.12. Although real lichens are not perfectly smooth, the corrugations and structures on a lichen's surface are irrelevant as long as the lichen remains close to a disc (compare figure 1*c* and inset). The use of equation (3.1) is thus justified and the total flux of carbon toward the lichen causes an equal increase in lichen mass, *m*:3.2
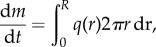
plugging equation (3.1) on the r.h.s. of equation (3.2) and using *m* = *πR*^2^*Hρ*_lichen_ with constant *H*, the radial growth rate of the lichen is3.3
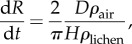
which is independent of lichen radius *R*. Equation (3.3) provides a direct formula for the expansion rate of maturely shaped lichens in terms of measurable parameters. Since smaller individuals are more rounded and grow slower, we refer to equation (3.3) as the maximum growth rate. With *D* = 16 mm^2^ s^−1^, an average density of carbon in the lichen *ρ*_lichen_ = 0.63 × 10^3^ kg m^−3^ ([21] and references therein), an average density of carbon in air *ρ*_air_ = *M*_c_*c*_CO_2__ = 2.1 × 10^−4^ kg m^−3^, and taking for example a reference lichen height of *H* = 4 mm, we can estimate3.4
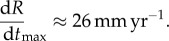
Lichens will rarely grow at this maximum growth rate because thalli are only photosynthetically active when habitats are the right combination of temperature, moisture, etc. and optimal environments may only occur sporadically. Photosynthesis will also be depressed when thalli are very wet because the diffusion of CO_2_ will slow within the water layer covering the thallus (e.g. [22,23]). To compare our model predictions with real growth speeds, we introduce an additional parameter *e* that represents the mean fraction of time a lichen is photosynthetically active (0 < *e* < 1):3.5
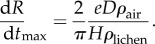
Of the parameters in this model, *D* and *ρ*_air_ are independent of the species and growth conditions and we assume *ρ*_lichen_ is also largely invariant. Hence the model predicts that maximum growth rates of different lichens will vary only when lichens differ in average height *H* or photosynthetic activity *e*.

## 4. Tests of the universal growth saturation

**Figure 2.**
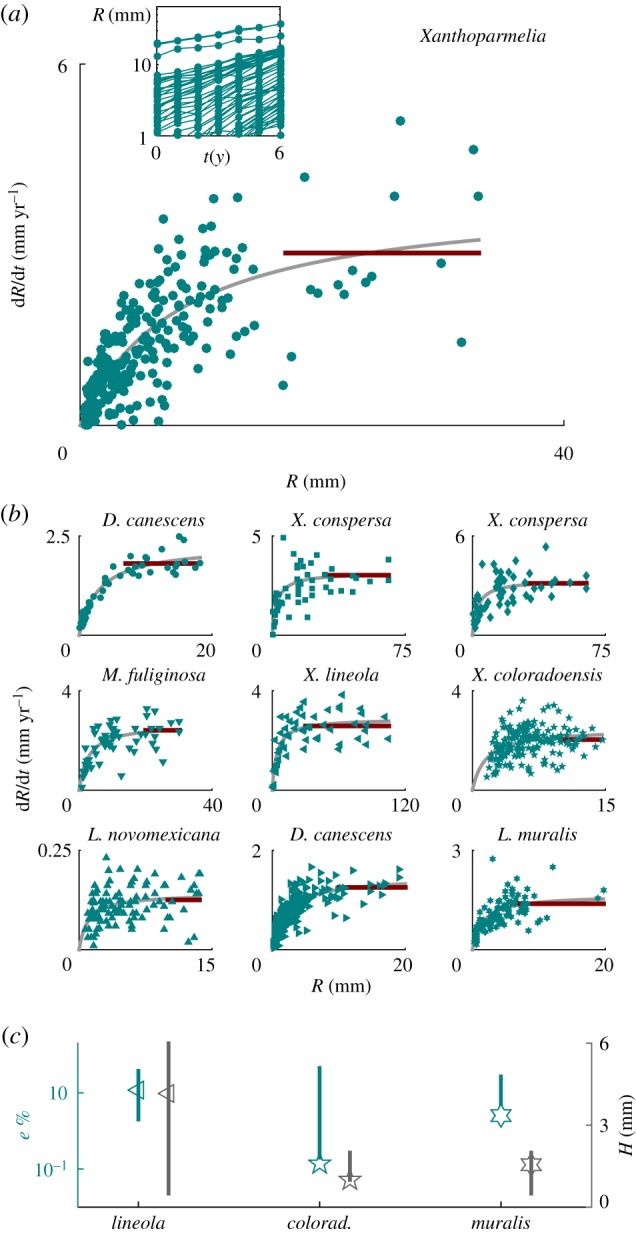
Experimental data of radial growth rates fit with the theoretical limit, equation (3.5), and with the functional form equation (4.1). (*a*) Data for *Xanthoparmelia* (this study, data provided as electronic supplementary material); inset: raw data for lichen radius as a function of time; (*b*) first row, left to right: *D. canescens* from [13]; *X. conspersa* from [26]; *X. conspersa* from [27]; second row: *M. fuliginosa* from [11]; *X. lineola* from [28]; *X. coloradoensis* from [29]; third row: *L. novomexicana* from [29]; *D. canescens* from [12]; *L. muralis* from [12]. In panels (*a*,*b*): cyan symbols are experimental data; the dark red line is the theoretical limit to growth caused by carbon dioxide flux, equation (3.5); the grey curve is the empirical growth curve connecting early and late growth regimes, equation (4.1). (*c*) Values of fitting parameters agree with independently collected experimental data. Symbols represent parameters obtained from best fits (grey curves in panels *a*,*b*) for *X lineanola*, *X. coloradoensis* and *L. muralis*; bars represent experimental measurements for the same parameters and their variation in nature from [11,30–32]. (Online version in colour.)

To quantify the saturation of the growth rate, we took advantage of our own direct measures of the growth of 53 individuals (figure 2*a*). Originally, a group of 55 foliose lichens growing on the French tombstone of the North Cemetery, Petersham, MA, USA (42°31′50.20′′ N; 72°11′22.19′′ W) was used to measure growth rates in nature. Target lichens are morphologically uniform and belong to the genus *Xanthoparmelia*. The taxonomy of species within *Xanthoparmelia* is controversial [24,25], and without genetic data we cannot assign a species epithet to the population. Inscribed letters and numbers were used to make a map of the entire population and identify individual thalli from year to year. Each thallus was measured each autumn for 7 years, starting in 2005. A transparent piece of plastic was placed over the thallus and the diameter of the thallus traced with permanent marker. Tracings were digitized and the area, *A*_*n*_, calculated from digitized images for each year *n*. Fifty-three new individuals born in 2006, 2007, 2008 and 2009 were added to the survey, and during the survey 33 individuals died. By the end of the survey, in 2011, data had been collected for a total of 75 individuals. Because our model concerns single, isolated, entire lichens, we discarded data for all lichens that fragmented (six individuals), merged with others (nine individuals) or where we noted possible disease or other kinds of damage during the period of observation (seven individuals). We retained a dataset of 53 lichens. For each individual, the growth rate d*R*/d*t* was determined as (*R*_*n*+1_ − *R*_*n*_)/1 year, where 
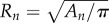
 is the calculated radius of the lichen in year *n*. Raw data for *R* versus *t* as well as d*R*/d*t* versus *R* are shown in figure 2*a* inset and main panel. While data were collected with care and rigor, scatter is an inevitable result of individual variation and environmental heterogeneity (i.e. the relative amounts of shade and sunlight at the top versus the bottom of a tall, columnar tombstone), and is an inherent feature of ecological data. We also identified nine published datasets on the growth rates of seven additional species; all growth rates were similarly measured in nature [11–13,26–29].

All data show saturation of the growth rate, as visualized by the placement of red, flat lines in figure 2. Data also provide evidence for a striking pattern of linear growth rate d*R*/d*t* ∼ *R* among smaller lichens. We use a simple functional form to interpolate between d*R*/d*t* ∼ *R* at small sizes and growth saturation d*R*/d*t* ∼ d*R*/d*t*_max_ at long timescales:4.1
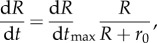
where *r*_0_ marks the transition between linear growth and growth saturation. We then fit equation (4.1) to the data (grey curves in figure 2*a*,*b*), which provides estimates for the two unknown parameters d*R*/d*t*_max_ and *r*_0_. If carbon dioxide flux is the factor limiting growth over the entire lifetime of a lichen, then we expect the transition to occur when the lichen becomes flat, i.e. when *R* grows larger than *H* (figure 1). Based on this prediction, we identify *r*_0_ ∼ *H* and using equation (3.5) our fitting parameters thus provide estimates for *e* and *H*. To test the consistency of the model, we next searched for published data which independently and experimentally measure the parameters *e* and *H* and found information for three of the seven species considered [11,27,30]. Our calculated values of the fitting parameters *H* and *e* are consistent with their natural range of variation, demonstrating consistency of the model and suggesting the model captures at least the essential elements of growth dynamics (figure 2*c*).

**Figure 3.**
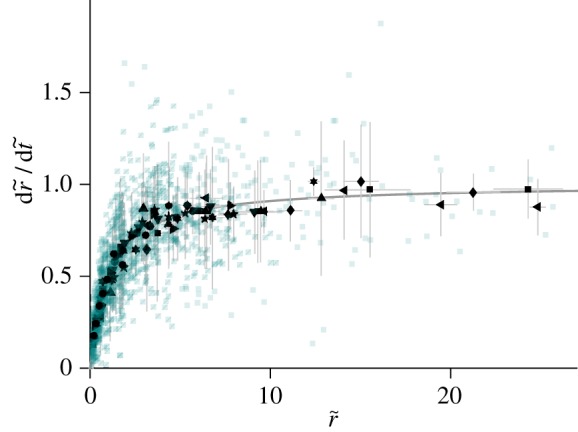
Empirical collapse of all datasets onto the same non-dimensional growth curve. The black solid line is the growth curve, equation (4.2) in terms of the non dimensional variables 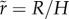 and 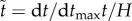, where *H* and d*R*/d*t*_max_ are obtained by fitting curves to data, see main text. Cyan squares represent all experimental data, both from this study and from the literature, see figure 2 caption. Black symbols and grey error bars are clustered averages and standard deviations for different species, symbols as in figure 2. (Online version in colour.)

Rescaling time and radius with 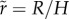 and 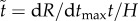 we can collapse all data onto the curve (figure 3):4.2
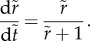
Data collapse is especially tight suggesting that the mechanism underlying growth kinetics is robust and applies to a vast number of crustose and foliose lichen species.

## 5. The growth rate for small lichens

**Figure 4.**
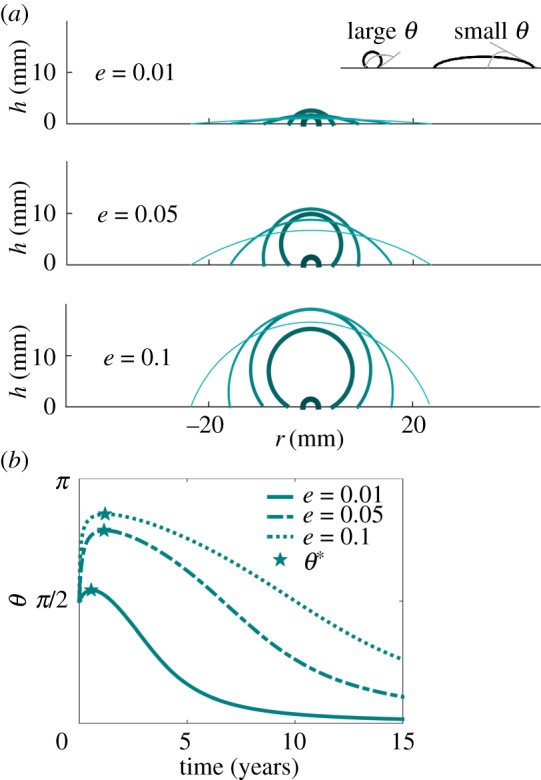
Lichen growth computed through equation (5.2), using data for *Xanthoparmelia* from this study for *R* and 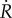; *D* = 16 mm^2^ s^−1^; *ρ*_lichen_ = 0.63×10^3^ kg m^−3^; *ρ*_air_ = 2.1×10^−4^ kg m^−3^; and three values for the parameter *e*. All simulations start from the same hemispherical initial condition with *R* = 1.5 mm. (*a*) Shape of the lichen. Time is coded from dark thick cyan to light thin cyan corresponding to snapshots after 0, 3, 6, 9 and 12 years. Higher values of *e* yield unrealistically thick lichens (A Pringle 2005–2007, personal observation), suggesting the most realistic scenario involves low values of *e*. Inset: sketch of contact angle. Rounded and flat lichens have large and small contact angles, respectively. (*b*) Evolution of contact angle in time, for different value of *e*. The lichen first quickly rounds up to a maximum contact angle θ^*^ that depends on *e* and the initial condition, and then slowly flattens out. (Online version in colour.)

We next explore the consequences of our hypothesis that the entire growth curve, including growth at early stages, is limited by carbon dioxide diffusion in the air. We first calculate the diffusive flux from equation (2.1) toward a small lichen, assuming its shape is approximately a spherical cap with contact angle *θ* and radius *R*. When the contact angle *θ* is large the lichen is round, and when *θ* is small the lichen is flat (see side view sketch in figure 4). This problem has been solved analytically in [19], with the result5.1
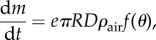
where 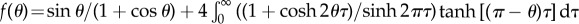. Equation (5.1) implies that the total flux of carbon dioxide toward the lichen translates to an increase in mass d*m*/d*t* of the spherical cap. Note that equation (5.1) asymptotes to equation (3.2) in the limit for small contact angle, i.e. for a disk. Using *m* = *πρ*_lichen_*R*^3^*g*(*θ*) where *g*(*θ*) = (cos^3^*θ* − 3 cos*θ* + 2)/(3 sin^3^*θ*) and after simple algebra we obtain the change through time of the contact angle *θ*:5.2
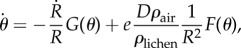
where *F* = *f*(*θ*)/*g*′(*θ*) and *G* = 3*g*(*θ*)/*g*′(*θ*). The negative term on the r.h.s. of equation (5.2) tends to flatten the lichen, whereas the positive term tends to round it up, making it closer to a sphere. Over a long period of time, growth saturates and the lichen's radius increases linearly in time according to equation (3.5): the prefactor of the positive term thus decreases ∼1/*t*^2^ faster than that of the negative term decreasing ∼1/*t*. Eventually the negative term takes over and the lichen flattens, confirming that growth saturation will occur when the lichen is flat. Before flattening, the shape of the lichen undergoes a transition the details of which depend on the exact value of parameters found in equation (5.2), the growth rates of individuals and the initial conditions.

**Figure 5.**
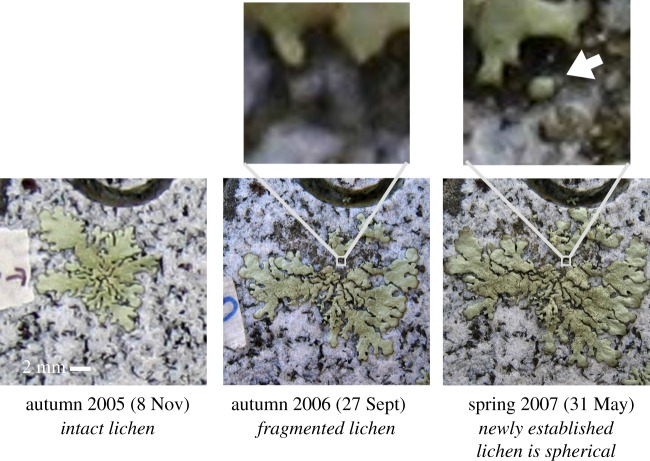
Newly established lichens are spherical. Photographs from a tombstone in the North Cemetery, Petersham, MA, USA. This individual was observed intensively as it established within a larger lichen and at early time points was nearly spherical. Note scale: the new individual measures less than 1/2 mm; new, tiny individuals are rarely observed or tracked in nature and data on shape are rare. (Online version in colour.)

To probe this transition we compute lichen shape over time by solving equation (5.2) using our own experimental data (figure 2*a*). Given *R*(*t*) from data, equation (5.2) predicts the dynamics of the contact angle, and using known values for *ρ*_lichen_, *ρ*_air_ and *D* (see caption of figure 4), we are left with a single non-dimensional parameter *e*. Figure 4*a* illustrates the shape dynamics of a *Xanthoparmelia* lichen starting from a hemisphere with radius *R*_0_ = 1.5 mm. We used three different values of *e* spanning one order of magnitude, chosen from within the known range of natural variation (figure 2*c*). Large values of *e* correspond to intense photosynthetic activity and consistently yield thicker lichens. Note that the lichen initially rounds up, quickly reaches a maximum contact angle, and then slowly flattens out (figure 4*b*). To verify the generality of this observation, we repeat the analysis with all 10 datasets (figure 2*b*). We use the value of *e* obtained from the fitting procedure described above and start with a hemispherical lichen of radius 1.5 mm as above. The results confirm that the lichens round up quickly before slowly flattening out, as observed for our own dataset. While the maximum contact angle depends on the initial conditions as well as the value of *e*, the transition is qualitatively robust. Interestingly, newly established lichens are in fact very round (A Pringle 2005–2007, personal observation). Figure 5 shows a photograph of a newly established lichen. However, exact and quantitative measurements of lichen shape over time are needed to fully corroborate our prediction.

## 6. Discussion

A simple physical model provides a universal growth limit relevant to tens of thousands of circular, crustose or foliose lichen species. While earlier models focused on internal carbon dynamics [12–14], ours is based only on the flux of carbon dioxide in the air above a lichen. Fundamental constraints on carbon dioxide diffusion are enough to predict observed patterns of growth. Our model clarifies the universal nature of growth limits: lichens cannot manipulate the diffusion of carbon dioxide in air, and differences among species in the efficiency of carbon transport within a thallus are irrelevant to a limit that is solely determined by large scale morphological features.

Other mechanisms will also influence the growth of lichens, and may be sources of error in our model. Growth may be modulated by aspects of algal or bacterial quality, supplies of scarce resources including nutrients scavenged from rainwater or substrates, competition with adjacent individuals, or disease. However, aspects of the model suggest the limiting factor for the growth of circular lichens is, in fact, the diffusive flux of carbon dioxide into a thallus. The growth limit is extremely fundamental and relies on just three basic assumptions: a boundary layer with nearly still air around a lichen, a thallus with a roughly disk-like morphology, and photosynthetic organisms capable of depleting carbon dioxide close to the surface of the thallus. We do not assume that photobionts at the lichen edge deplete carbon dioxide more quickly than photobionts at the centre. When these three assumptions are met, the limit we describe in equation (3.5) has to hold, independently of any other mechanisms or differences among species. Using a typical thallus height of 4 mm, the carbon density of lichens, and the material properties of surrounding air as variables, our prediction of the maximum possible growth speed is about 26 mm yr^−1^, and is surprisingly close in magnitude to the fastest reported growth speeds, e.g. 13 mm yr^−1^ for a species of *Parmelia* [11,33]. Moreover, the model generates several general and easily verifiable (or falsifiable) predictions: (i) the transition from d*R*/d*t* ∼ *R* and d*R*/d*t* ∼ constant will depend purely on geometry, and will happen after a lichen's radius becomes greater than its height (ii) values for average height *H* and photosynthetic activity *e* can be found by fitting growth data (as measured by changes in radius) to equation (4.1) (iii) for large lichens radial growth is caused primarily by photosynthesis at the lichen's edge. Note that (iii) is not an assumption, but a prediction of the model, as it follows directly from the idea that lichen growth is limited by carbon dioxide uptake. Note also that we do not consider reproduction in our model. We speculate that any photosynthates generated at the centres of larger lichens are disproportionately used to grow reproductive structures, which are often concentrated towards the centre of a thallus and in turn may affect the height and roughness of an individual.

Lichens are not easy to grow in the laboratory and most data are taken in nature. But available field data are inherently noisy; variability likely stems from intraspecific variability, differences in the external environments around individual lichens, and the challenges of accurately recording growth rates of very small or slow growing thalli. The mechanistic principles we offer may also emerge as a useful guide to data fitting; although the general trend is for the growth rate of large lichens to saturate, often several curves can be used to fit the same data [6]. And while tests of the available hypotheses used to explain lichen growth remain challenging, our model suggests various routes forward.

The model's predictions can be tested with experiments, as can hypotheses associated with our analysis of small lichens. Any data that measure radial growth speed would test predictions associated with the growth limit equation (3.5), and independent measures of *H* and *e* would validate the fit of growth rate data to equation (4.1); if the entire three-dimensional shape of the growing lichen was also measured, including the contact angle *θ*, data would verify (or reject) the entirety of our theory. Aspects of geometry (*R*, *H* and *θ*) might be measured directly, while average photosynthetic activity can be measured with tools targeting isotopes or gas exchange. Testing whether the edges of a lichen are highly photosynthetically active, compared to its centre, might involve chlorophyll fluorescence imaging to generate a highly resolved spatial map of photosynthesis. The variations of *H* among individuals of a species can be large, because of the intrinsic variability of environments and individuals, and in part because of reproductive dynamics. Available data for *H* and *e* are snapshots, often from a single individual or single point in time. Rigorous tests of our model would require multiple measures of both *H* and *e* for many individuals in multiple habitats.

The predicted growth pattern depends on a small boundary layer of static air above the lichen surface, and diffusion within the boundary layer. While these assumptions will generally be met in the field, conditions might be modified in the laboratory, if entire lichens were moved indoors. Forcing constant, highly unsteady flow over a lichen surface would make advection dominate over diffusion and in these environments the predicted growth law would disappear.

The model may also explain other features of lichen growth which have already been observed in nature and during experiments. For example, the centre of a thallus often falls out when a lichen is sufficiently large, although the growth rate of the thallus at its edges remains unchanged [11]. Lichens without centres grow as quickly as control lichens with intact centres [34], and shading everything but the small band at the edge of a thallus also has no effect on growth [11]. Our model predicts that as a lichen gets older and larger, the flux of carbon dioxide into the centre of a thallus decreases and eventually becomes negligible: the lack of carbon may cause the centre of a lichen to fall apart or die. Moreover, because most carbon dioxide intake is concentrated at lichen edges, the centre of the lichen will have no direct impact on peripheral growth, explaining why the growth rates of edges remain constant even when centres are missing. The model and available data may also explain why experiments designed to track the movement of carbon within lichens are unsuccessful [11]. Carbon influx is greatest at lichen edges, and this influx drives growth; there may be no movement of carbon within the thallus. In fact carbon dioxide flux in the air above a lichen is at the origin of the growth pattern in our model, and so our thinking is profoundly different from the assumptions of previous models, which focus on the movement of carbon within a thallus and assume transport will shape growth dynamics.

Finally, the mechanism we identify as underpinning lichen growth may be relevant to the growth of other microorganisms with a similar geometry. The canonical example is the growth of bacterial colonies on a Petri dish, epitomized by *Bacillus subtilus* [35]. The geometry of a bacterial colony is similar to the geometry of a circular lichen; a bacterial colony grows out from a centre as a disk over a Petri dish. As with lichens, as the colony grows, there are changes at the centre of the colony [36,37]: in the bacterial colony a bistable switch causes cells to transition from being motile and possessing flagella to expressing extracellular matrix [38]. The first cells that express matrix are within the centre of the colony whereas the motile cells remain on the outside. As the colony develops further there is another transition at the centre of the colony, to sporulation. Intriguingly, the mechanism for the growth of a *Bacillus* colony is similar to the mechanism we describe here, but instead of the diffusion of carbon dioxide, the growth of the bacterial colony is limited by the diffusion of nutrients in the agar [39]. As with the lichen, but from the standpoint of the nutrient diffusion problem in the agar, the colony is a perfect absorber. In other words the mathematical framework that we describe translates directly to bacterial growth. Equation (3.3) predicts the relationship between the colony growth rate and the diffusivity of nutrients, the height of the colony and the relative concentrations of nutrients and bacteria. Using parameters for *Bacillus subtilis* biofilms (see [40] and references therein), diffusivity of glycerol *D* = 5 × 10^−4^ mm^2^ s^−1^; density of carbon in the medium *ρ*_agar_ = 1.8 × 10^−4^ g cm^−3^, density of carbon in the cell *ρ*_cell_ = 0.18 g cm^−3^; biofilm thickness *H* = 0.15 mm and volume fractions from 0.2 to 0.6, we obtain a maximum growth rate 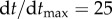
–75 μm h^−1^, comparable with typically observed growth rates of 140 μm h^−1^. As the colony grows, nutrient concentrations drop in the centre of the colony [40]. Laplace equation (2.1) describes how when the colony grows the nutrient flux drops in the centre of the colony; this nutrient depletion has been demonstrated to cause the bistable switch. A quantitative comparison of these features with bacterial growth dynamics is beyond the scope of our current manuscript but remains an intriguing direction for future research.

Our model highlights the subtle roles physics and fluid dynamics can take to shape the growth and morphologies of organisms. Constraints on carbon dioxide diffusion may also limit the growth of lichens with other body plans, including shrubby, fruticose lichens. But whether constraints do control the growth of lichens with more complex morphologies remains an open, and fascinating, question.

## Supplementary Material

Data collected in this study and used for analysis (Figure 2A and inset)

## Data accessibility

Data collected for this study (figure 2*a*) are accessible as supplementary material; all other data (figure 2*b*) have been obtained from the referenced literature.

## References

[1] Brodo IM, Sharnoff SD, Sharnoff S 2001 Lichens of North America, 1st edn New Haven, CT: Yale University Press.

[2] Mushegian AA, Peterson CN, Baker CCM, Pringle A 2011 Bacterial diversity across individual lichens. Appl. Environ. Microbiol. 77, 4249–4252. (10.1128/AEM.02850-10) 21531831 PMC3131627

[3] Ding L, Zhou Q, Wei J 2013 Estimation of *Endocarpon pusillum* Hedwig carbon budget in the Tengger Desert based on its photosynthetic rate. Sci. China - Life Sci. 56, 848–855. (10.1007/s11427-013-4526-9) 23907293

[4] Lange OL, Meyer A, Zellner H, Heber U 1994 Photosynthesis and water relations of lichen soil crusts: field measurements in the coastal fog zone of the Namib Desert. Funct. Ecol. 8, 253–264. (10.2307/2389909)

[5] Armstrong RA 1974 Growth phases in the life of a lichen thallus. New Phytol. 73, 913–918. (10.1111/j.1469-8137.1974.tb01320.x)

[6] Trenbirth HE, Matthews JA 2010 Lichen growth rates on glacier forelands in Southern Norway: preliminary results from a 25-year monitoring programme. Geografiska Annaler Ser. A-Phys. Geogr. 92A, 19–39. (10.1111/j.1468-0459.2010.00375.x)

[7] Innes JL 1985 Lichenometry. Prog. Phys. Geogr. 9, 187–254. (10.1177/030913338500900202)

[8] Bull WB, Brandon MT 1998 Lichen dating of earthquake-generated regional rockfall events, Southern Alps, New Zealand. Geol. Soc. Am. Bull. 110, 60–84. (10.1130/0016-7606(1998)110%3C0060:LDOEGR%3E2.3.CO;2)

[9] Adriana Garibotti I, Villalba R 2009 Lichenometric dating using *Rhizocarpon* subgenus *Rhizocarpon* in the Patagonian Andes, Argentina. Quat. Res. 71, 271–283. (10.1016/j.yqres.2009.01.012)

[10] Loso MG, Doak DF 2006 The biology behind lichenometric dating curves. Oecologia 147, 223–229. (10.1007/s00442-005-0265-3) 16237538

[11] Armstrong RA, Bradwell T 2011 Growth of foliose lichens: a review. Symbiosis 53, 1–16. (10.1007/s13199-011-0108-4)

[12] Hill DJ 1981 The growth of lichens with special reference to the modelling of circular thalli. Lichenologist 13, 265–287. (10.1017/S0024282981000352)

[13] Aplin PS, Hill DJ 1979 Growth analysis of circular lichen thalli. J. Theor. Biol. 78, 347–363. (10.1016/0022-5193(79)90335-7) 513787

[14] Childress S, Keller JB 1980 Lichen growth. J. Theor. Biol. 82, 157–165. (10.1016/0022-5193(80)90095-8) 7401656

[15] Palmqvist K 2000 Carbon economy in lichens. New Phytol. 148, 11–36. (10.1046/j.1469-8137.2000.00732.x) 33863029

[16] Deegan RF, Bakajin O, Dupont TF, Huber G, Nagel SR, Witten TA 1997 Capillary flow as the cause of ring stains from dried liquid drops. Nature 389, 827–829. (10.1038/39827)

[17] Ryan BD, Bungartz F, Nash TH III 2002 Morphology and anatomy of the lichen thallus. In Lichen Flora of the greater Sonoran Desert region (eds Nash TH III, Ryan BD, Gries C, Bungartz F), pp. 8–23. Tempe, AZ: Lichens Unlimited.

[18] Holstag AAM, Van Ulden AP 1983 A simple scheme for daytime estimates of the surface fluxes from routine weather data. J. Clim. Appl. Meteorol. 22, 517–529. (10.1175/1520-0450(1983)022%3C0517:ASSFDE%3E2.0.CO;2)

[19] Popov YO 2005 Evaporative deposition patterns: spatial dimensions of the deposit. Phys. Rev. E 71, 036313 (10.1103/PhysRevE.71.036313) 15903580

[20] Jackson JD 2001 Classical electrodynamics, 3rd edn New York, NY: Wiley.

[21] Holt R, Moen J, Danell O 2007 Non-destructive estimation of lichen biomass. Math. Syst. Theory 27, 41–47. (10.7557/2.27.1.172)

[22] Lange OL, Tenhunen JD 1981 Moisture content and CO_2_ exchange of lichens. II. Depression of net photosynthesis in *Ramalina maciformis* at high water content is caused by increased thallus carbon dioxide diffusion resistance. Oecologia 51, 426–429. (10.1007/BF00540917) 28310031

[23] Cowan IR, Lange OL, Green TGA 1992 Carbon-dioxide exchange in lichens: determination of transport and carboxylation characteristics. Planta 187, 282–294. (10.1007/BF00201952) 24178057

[24] Leavitt SD, Johnson L, St Clair LL 2011 Species delimitation and evolution in morphologically and chemically diverse communities of the lichen-forming genus *Xanthoparmelia* (Parmeliaceae, Ascomycota) in western North America. Am. J. Bot. 98, 175–188. (10.3732/ajb.1000230) 21613107

[25] Thorsten Lumbsch H, Leavitt SD 2011 Goodbye morphology? A paradigm shift in the delimitation of species in lichenized fungi. Fungal. Divers. 50, 59–72. (10.1007/s13225-011-0123-z)

[26] Armstrong RA 1992 A comparison of the growth curves of the foliose lichen *Parmelia conspersa* determined by a cross-sectional study and by direct measurement. Environ. Exp. Bot. 32, 221–227. (10.1016/0098-8472(92)90005-M)

[27] Armstrong RA, Smith SN 1996 Factors determining the growth curve of the foliose lichen *Parmelia conspersa*. New Phytol. 134, 517–522. (10.1111/j.1469-8137.1996.tb04369.x)

[28] Benedict JB, Nash TH 1990 Radial growth and habitat selection by morphologically similar chemotypes of *Xanthoparmelia*. Bryologist. 93, 319–327. (10.2307/3243520)

[29] Benedict JB 2008 Experiments on lichen growth. III. The shape of the age–size curve. Arctic Antarctic Alpine Res. 40, 15–26. (10.1657/1523-0430(06-030)%5BBENEDICT%5D2.0.CO;2)

[30] Armstrong RA 2011 The biology of the crustose lichen *Rhizocarpon geographicum*. Symbiosis 55, 53–67. (10.1007/s13199-011-0147-x)

[31] Lange OL 2003 Photosynthetic productivity of the epilithic lichen *Lecanora muralis*: long-term field monitoring of CO_2_ exchange and its physiological interpretation: II. Diel and seasonal patterns of net photosynthesis and respiration. Flora - Morphol. Distrib. Funct. Ecol. Plants 198, 55–70. (10.1016/S0367-2530(04)70052-3)

[32] Benedict JB 1990 Experiments on lichen growth. I. Seasonal patterns and environmental controls. Arctic Antarct. Alpine Res. 22, 244–254. (10.2307/1551587)

[33] Paulson R 2006 Notes on the ecology of lichens with special reference to Epping Forest. Essex Nat 18, 276–286.

[34] Armstrong RA 1979 Growth and regeneration of lichen thalli with the central portions artificially removed. Environ. Exp. Bot. 19, 175–178. (10.1016/0098-8472(79)90046-7)

[35] López D, Vlamakis H, Kolter R 2010 Biofilms. Cold. Spring. Harb. Perspect. Biol. 2, a000398 (10.1101/cshperspect.a000398) 20519345 PMC2890205

[36] Wang X *et al.* 2016 Probing phenotypic growth in expanding *Bacillus subtilis* biofilms. Appl. Microbiol. Biotechnol. 100, 4607–4615. (10.1007/s00253-016-7461-4) 27003268

[37] Wilking JN, Zaburdaev V, De Volder M, Losick R, Brenner MP, Weitz DA 2013 Liquid transport facilitated by channels in *Bacillus subtilis* biofilms. Proc. Natl Acad. Sci. USA 110, 848–852. (10.1073/pnas.1216376110) 23271809 PMC3549102

[38] Kearns DB, Chu F, Branda SS, Kolter R, Losick R 2005 A master regulator for biofilm formation by *Bacillus subtilis*. Mol. Microbiol. 55, 739–749. (10.1111/j.1365-2958.2004.04440.x) 15661000

[39] Seminara A, Angelini TE, Wilking JN, Vlamakis H, Ebrahim S, Kolter R, Weitz DA, Brenner MP 2012 Osmotic spreading of *bacillus subtilis* biofilms driven by an extracellular matrix. Proc. Natl Acad. Sci. USA 109, 1116–1121. (10.1073/pnas.1109261108) 22232655 PMC3268299

[40] Zhang W, Seminara A, Suaris M, Brenner MP, Weitz DA, Angelini TE 2014 Nutrient depletion in *Bacillus subtilis* biofilms triggers matrix production. New. J. Phys. 16, 015028 (10.1088/1367-2630/16/1/015028)

